# Equivalent consumption minimization strategy based on global optimization of equivalent factor for hybrid tractor

**DOI:** 10.1038/s41598-024-63770-w

**Published:** 2024-06-05

**Authors:** Zhen Zhu, Yihan Zhang, Hongwei Zhang, Dehai Wang, Long Chen

**Affiliations:** 1 Automotive Engineering Research Institute, Jiangsu University, Zhenjiang, 212013 Jiangsu China; 2 National Key Laboratory of Special Vehicle Design and Manufacturing Integration Technology, Baotou, 014030 Inner Mongolia China; 3 Laboratory of Fluid Power and Mechatronic Systems, Zhejiang University, Hangzhou, 310027 Zhejiang China

**Keywords:** Plug-in hybrid tractor, Genetic algorithm, Equivalent factor optimization, Energy management, Energy science and technology, Mechanical engineering

## Abstract

Due to the increase in emission requirements for non-road vehicles in many countries and the reduction of agricultural personnel, tractors are developing towards high horsepower and electrification. According to the working conditions of high-horsepower tractors, a hydromechanical continuously variable transmission (HMCVT) is designed for hybrid tractors. Taking a tractor equipped with this transmission as the research object, an equivalent factor global optimization model was established and a genetic algorithm was used to optimize the equivalent factor *S* offline to obtain the optimal equivalent factor of the tractor under different operating mileage and the initial state of charge (SOC) of battery. By using the optimized equivalent factor, the tractor can be in the charge depleting (CD) mode for a longer time on the premise of making full use of the energy in the battery, so as to improve the auxiliary ability of the motor in the whole operation cycle to reduce the fuel consumption of the tractor. The effectiveness of the control strategy is verified by MATLAB/Simulink and hardware in the loop (HIL) tests, and the fuel economy of tractors is improved by 2.939% and 3.909% respectively in the two tests.

## Introduction

Since the 14th Five-Year Plan, our country has vigorously promoted the integrated application of continuously variable speed, new energy power, mechanical–electrical-hydraulic integration and other technologies in agricultural machinery and equipment and accelerated the innovative development of large-scale high-end intelligent agricultural machinery and equipment^1^. With the aging of population and urbanization leading to the reduction of agricultural population and the intensification of farmland, the demand for high-power agricultural machinery is further increased^2,3^. Traditional diesel tractors have problems of poor fuel economy and serious exhaust pollution. Limited by the development of batteries, pure electric tractors have problems such as poor endurance and slow energy replenishment, which make it difficult to meet the needs of continuous high-power operations. Hybrid tractors introduce an electric motor as an auxiliary power source in addition to the diesel engine, which can prevent the diesel engine from operating in a low-efficiency range for a long time and realize energy recovery during braking, thereby reducing energy consumption. Therefore, hybrid power is considered an ideal solution to reduce the energy consumption of high-horsepower tractors^4^.

In order to obtain good fuel economy of tractors, the power distribution among power sources needs to be coordinated by control strategy^5^, and the power transmission route also needs to be allocated by control strategy^6^. Currently, the rule-based energy management strategy is widely used because of its small amount of calculation and high reliability^7^, but it also heavily depends on the experience and professional knowledge of developers, but it also depends heavily on the experience and professional knowledge of developers, and because the control framework is relatively simple, it also makes it difficult to achieve targeted dynamic adjustment in complex working environment, so it is difficult to give full play to the energy-saving advantages of plug-in hybrid tractors. The learning-based energy management strategy can improve the control effect of the system without establishing a clear mathematical model or solving algorithm, but the learning-based energy management strategy depends on a reasonable reward function and a large number of training samples. It is difficult to guarantee its performance, so it still needs further research and verification^8^. Therefore, the control strategy based on optimization is the main research object at present, and the equivalent consumption minimum strategy has been gradually applied in the engineering field because of its good real-time^9^.

Zhang ^10^ proposed a CD-CS based control strategy, which adopts a rule-based strategy in the charge depleting stage and an ECMS control strategy in the Charge Sustaining (CS) stage to improve the fuel economy of plug-in hybrid vehicles. Lin Mengyou et al.^11^ combined the variable equivalent factor ECMS control strategy with CD-CS control to propose an equivalent factor discrete global optimization equivalent fuel instantaneous consumption minimum strategy energy management strategy. Compared with the strategy before optimizing the equivalent factor, the fuel economy is improved by 20.81%. Yang et al.^12^ puts forward a kind of Fuzzy PA-ECMS and Fuzzy MPGA-ECMS applied to double planetary gear hybrid city bus. Compared with the traditional rule-based control strategy, the fuel economy of the two is improved by 5.29% and 5.31% respectively. Ngoc-LamVu et al.^13^ proposed a rule-based energy management strategy suitable for hybrid tractors equipped with snow plows, which enables the efficiency of most operating points of the tractor to reach 85%-87%, making the tractor fuel consumption reduced by 33.4%.

Since the average commuting distance of mega-city is 9.4 km, and that of Type II city is 7.6 km^14^. Therefore, plug-in hybrid passenger cars generally consider short-distance travel more. When the battery power is sufficient, the priority of using the motor as the power source is very high, and the engine will output only when the battery SOC is low or the motor power can not meet the driving requirements of the vehicle. In many cases, hybrid high-power tractors may need to be operated for more than 8 h per day^15^, but the current battery technology is difficult to meet the motor to drive high-power tractors for a long time, so the motors of hybrid tractors are usually used for auxiliary drive. Currently, the energy management of hybrid tractors is studied by teachers, while the control strategy of plug-in passenger cars is not suitable for plug-in hybrid tractors. To further improve the auxiliary driving ability of the motor, it is necessary to plan the battery consumption speed of the tractor in the CD phase. For this reason, this paper combines CD-CS strategy with ECMS strategy to control the decline rate of battery SOC of plug-in hybrid tractor in CD stage. the main contents are as follows: the global optimization model of equivalent coefficient of plug-in hybrid tractor is established, and the genetic algorithm is used to optimize the equivalent coefficient off-line according to the working conditions of tractor, and the optimal equivalent coefficient under different initial SOC and driving mileage is obtained. Furthermore, the ECMS strategy is used to optimize the power distribution of the tractor according to the optimal equivalent coefficient, so that the motor can better assist the diesel engine in the working cycle, thus improving the fuel economy of the plug-in hybrid tractor.

## Power system structure and operating conditions

### Power system structure

At the present stage, high-horsepower tractors mostly use mechanical transmission combined with diesel engine for power transmission. However, mechanical transmissions are difficult to achieve continuously variable speed under high loads, causing the diesel engines to work in a low-efficiency range for a long time. Therefore, a hydro-mechanical continuously variable transmission suitable for plug-in hybrid tractor is designed to improve the working efficiency of high horsepower tractor.

**Figure 1:**
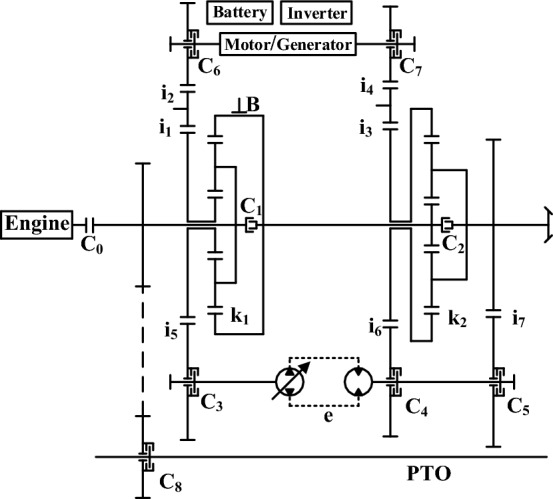
Structural schematic diagram of power transmission system of plug-in hybrid tractor.

Figure 1 shows the power system of the plug-in hybrid tractor studied in this paper. Its power transmission system mainly includes WP6.180E40 diesel engine, power battery, TZ205XS85K01 motor, main reducer, hydraulic mechanical continuously variable transmission and so on. In this system, the switching of driving mode is realized by controlling the state of clutch and brake, and the transmission ratio of transmission is controlled by adjusting the displacement ratio of hydraulic transmission. The working stage of the tractor is mainly divided into CD stage and CS stage, and the two stages are sub-divided according to different load conditions.

Taking Dongfanghong LD-1804 wheeled tractor as a reference vehicle, a plug-in hybrid tractor model equipped with this power system is created in MATLAB/Simulink. The main parameters of the model are shown in Table 1.

**Table 1:** The main parameters of the model.

Parts	Parameters	Value
Vehicle	Vehicle mass/kg	8200
	Driving wheel radius / m	0.875
Engine	Rated power/kW	132
	Rated speed/r∙min^-1^	2300
	Maximum torque/Nm	750
Motor	Maximum power/kW	85
	Rated power/kW	45
	Maximum torque/Nm	250
	Rated torque/Nm	130
Battery	Rated capacity/Ah	300
	Rated voltage/V	366
Transmission system	HMCVT deceleration ratio	1.00–3.57
	Main deceleration ratio	3.70
	Wheel edge deceleration ratio	6.40

### Transmission modelling

**Figure 2:**
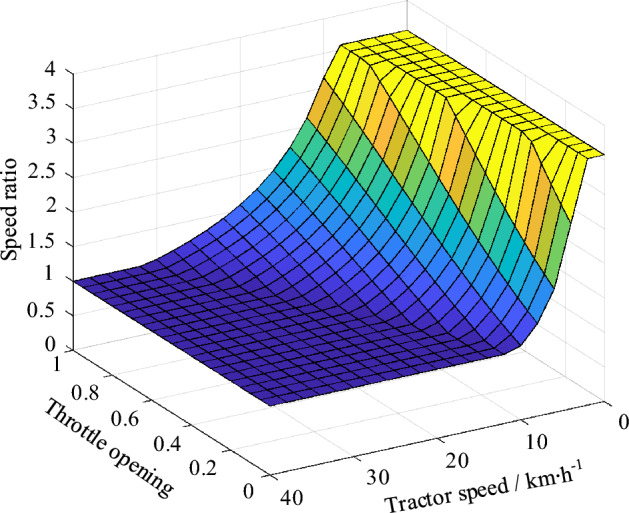
Optimal economic velocity ratio surface of HMCVT.

According to the data obtained from the bench test, the relationship between the optimal economic speed ratio and throttle opening of diesel engine is obtained. Since the theoretical non-sliding slope of the tractor on the cultivated land, clayey soil and sandy loam is more than 25°, $\delta$ can take 10%^17^, combined with the relationship between the speed of the hybrid tractor and the HMCVT transmission ratio, the optimal economic speed ratio surface of the hydraulic mechanical CVT shown in Fig. 2 can be obtained.

1 $$\left\{ \begin{gathered} n_{e\_opt} = - 210\alpha^{4} - 150\alpha^{3} + 810\alpha^{2} + 1000\alpha + 750 \hfill \\ v_{a} = \frac{{0.377n_{engine} R_{wheel} (1 - \delta )}}{{i_{CVT} i_{main} i_{wheel} }} \hfill \\ \end{gathered} \right.$$

where $n_{e\_opt}$ is the optimal economic speed of the diesel engine; $\alpha$ is the throttle opening; $n_{engine}$ is the engine diesel speed; $R_{wheel}$ is the driving wheel radius of the tractor; $i_{CVT}$ is the transmission ratio of HMCVT; $i_{main}$ is the transmission ratio of main reducer; $i_{wheel}$ is the transmission ratio of wheel-side reducer; $\delta$ is the slip rate of the driving wheel.

### Working condition setting

Deutsche Landwirtschafts-Gesellschaft sets 14 test conditions according to the actual operating requirements of tractors, including 4 heavy-duty operating conditions with 100% load, 5 medium-load operating conditions with 60–70% load, 3 light-load operating conditions with 40% load, 1 heavy-load transport condition with 100% load and 1 light-load transport condition with 25% load^18^.The US Environmental Protection Agency has drawn up 16 transient working cycles for off-road vehicles^19^.

**Figure 3:**
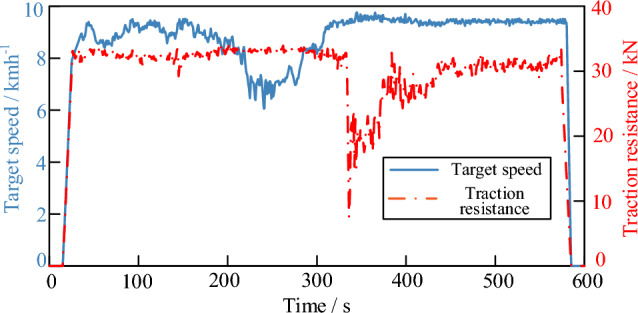
Working condition of hybrid tractor.

Since high-horsepower tractors is less likely to be transported, the speed cyclic conditions of the hybrid tractor under operating conditions was established by combining the test standards of the two organizations and the design driving speed of the hybrid tractor under ploughing operation. The traction resistance of the tractor during ploughing operation is affected by a variety of factors, so the ploughing operation test was conducted at a ploughing depth of 0.26 m and the traction resistance data were obtained using a tension sensor, and then the traction resistance cycle conditions were obtained. The speed cyclic conditions and traction resistance cyclic conditions shown in Fig. 3.

## Equivalent factor optimization model

### Optimization algorithm of equivalent factor

ECMS strategy converts the electric power consumption of the vehicle into fuel consumption by introducing the equivalent factor and takes the minimum equivalent total fuel consumption of the vehicle at each time as the optimization objective for optimal control^20,21^. The mathematical model can be calculated as follows.

2 $$\mathop m\limits_{equal} (t) = \mathop m\limits_{fuel} (t) + S(t)\frac{{P_{Motor} (t)}}{q}$$

where $\mathop m\limits_{equal} (t)$ is the equivalent fuel consumption rate of the whole vehicle at time $t$; $\mathop m\limits_{fuel} (t)$ is the fuel consumption rate of the diesel engine at the time $t$; $S(t)$ is the equivalent coefficient at time $t$; $P_{Motor} (t)$ is the power of the motor at time $t$; $q$ is the lower calorific value of diesel, which is taken as 42705 kJ/kg.

The Eq. is solved by Pontriagin's minimum principle, and the canonical equations of Hamiltonian function and Lagrange multiplier are as follows:

3 $$\left\{ \begin{gathered} H(SOC,t) = \mathop m\limits_{fuel} (t) + \lambda (t)\mathop {SOC}\limits (t) \hfill \\ \mathop \lambda \limits (t) = - \lambda (t)\frac{{\partial \mathop {SOC}\limits (t)}}{\partial SOC(t)} \hfill \\ \end{gathered} \right.$$

where $\lambda (t)$ is the Lagrange multiplier at the time $t$; $SOC(t)$ is battery SOC at the time $t$.

For plug-in hybrid tractors, the most important thing of ECMS control strategy is to find an optimal sequence of equivalent factors, and then to achieve the optimal power distribution n of motor and diesel engine to obtain the lowest operating cost. Because of the large randomness of $\lambda (t)$ and the need to predict the required power in advance to obtain the best $\lambda (t)$ , this is difficult to achieve in the actual operation process. However, the variation trend of equivalent factor $S$ with soc value is known. When the value of battery SOC is higher, the equivalent coefficient $S$ is smaller, which makes the bias strategy more inclined to use the motor as the power source, while when the value of battery SOC is lower, the value of equivalent coefficient $S$ is larger, which makes the control strategy more inclined to use the diesel engine as the power source. According to the relative relationship between the equivalent factor $S$ and the battery SOC, an initial penalty function is established, and then the correction coefficient is used to adjust the function.

To perform offline optimization of the equivalent coefficient under different initial SOC values ​​and driving mileage, a correction coefficient is introduced to adjust the penalty function to obtain the best equivalent coefficient. The equivalent factor optimization model can be established as follows.

4 $$S_{opt} (SOC) = kf(SOC)$$

where $S_{opt}$ is the optimal equivalence coefficient; $k$ is Correction factor; $f(SOC)$ is initial penalty function whose value is determined by the lookup table .When the value of battery SOC is large (about 0.9), the function value of the basic penalty function is about 0.5; with the decrease of the battery SOC, the function value of the basic penalty function increases slowly; when the value of battery SOC decreases to 0.25, the function value of the basic penalty function increases to 0.6; after that, the function value of the basic penalty function increases rapidly with the decrease of the battery SOC.

### Offline global optimization of equivalent factors

According to the equivalent factor optimization model established in Section "Optimization algorithm of equivalent factor", the global optimization model of equivalent factor S is established, and its cost function and system constraints are shown in Eq. (5).

5 $$\left\{ \begin{gathered} {\text{L}}_{k} = \min_{{\left\{ k \right\}}} \int_{0}^{t} {\min_{{\{ P_{engine} ,P_{motor} \} }} \mathop {m_{equal} }\limits } dt \hfill \\ P_{engine\_\min } \le P_{engine} (t) \le P_{engine\_\max } \hfill \\ P_{motor\_\min } \le P_{motor} (t) \le P_{motor\_\max } \hfill \\ SOC_{\min } \le SOC \le SOC_{\max } \hfill \\ \end{gathered} \right.$$

where $P_{engine\_\max }$ and $P_{engine\_\min }$ represent the maximum and minimum output power of the diesel engine respectively; $P_{motor\_\max }$ and $P_{motor\_\min }$ represent the maximum and minimum output power of the motor, respectively; $SOC_{\max }$ and $SOC_{\min }$ are the maximum and minimum values of the battery SOC respectively.

Under the set working conditions, the power distribution of the diesel engine and motor is adjusted by adjusting the value of the correction coefficient to minimize the value of the cost function. In this way, the sequence of the optimal correction coefficient is obtained, and then the optimal equivalent coefficient sequence can be calculated by Eq. (4).

Because the global optimization of the equivalent factor is a nonlinear global optimization, genetic algorithm is chosen to optimize the equivalent factor. Genetic algorithm imitates the mechanism of biological evolution and searches through mutation, crossover, and selection to find the optimal solution or approximate optimal solution of system optimization.

**Figure 4:**
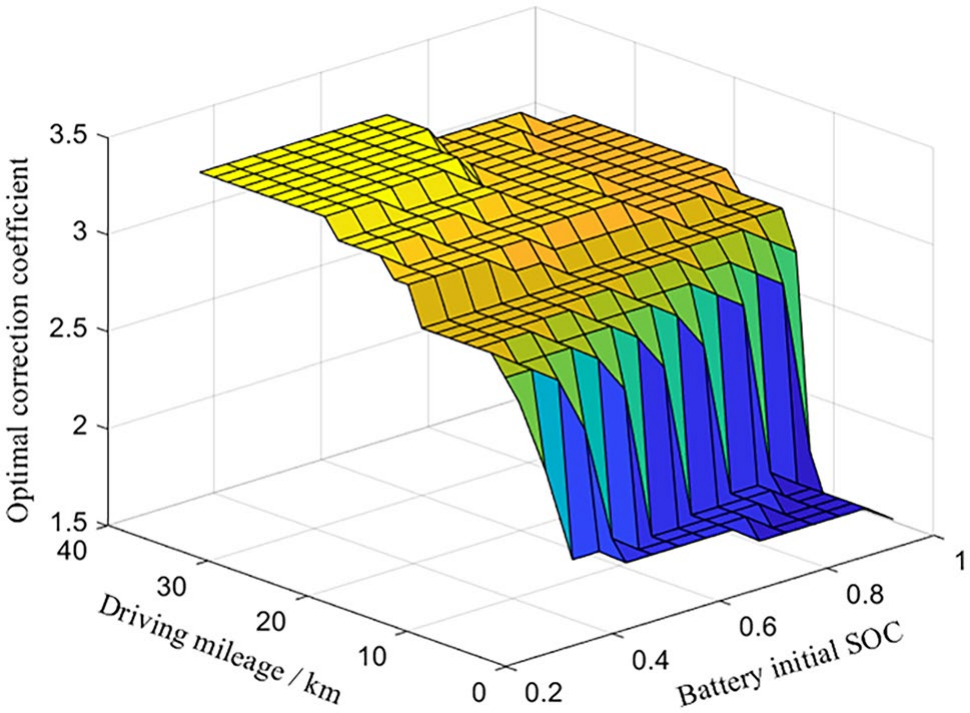
The optimum correction factors for different mileage and battery initial SOC.

The algorithm takes the fuel economy of the vehicle under the above operating conditions as the evaluation index, the number of iterations is 100, the number of populations is 64, the number of elites is 10, the value of crossover ratio is 0.2 and the value of variation ratio is 0.2. Under different initial values of battery SOC and mileage, the offline global optimization of the correction coefficient is carried out, and the optimal correction coefficient MAP shown in Fig. 4 is obtained. It can be seen from Fig. 4 that when the driving mileage is constant, the optimal correction coefficient decreases with the increase of the initial value of the battery SOC, indicating that when the battery has sufficient power, the efficiency of using electric energy is higher than that of using fuel.

## ECMS based on equivalent factor optimization

**Figure 5:**
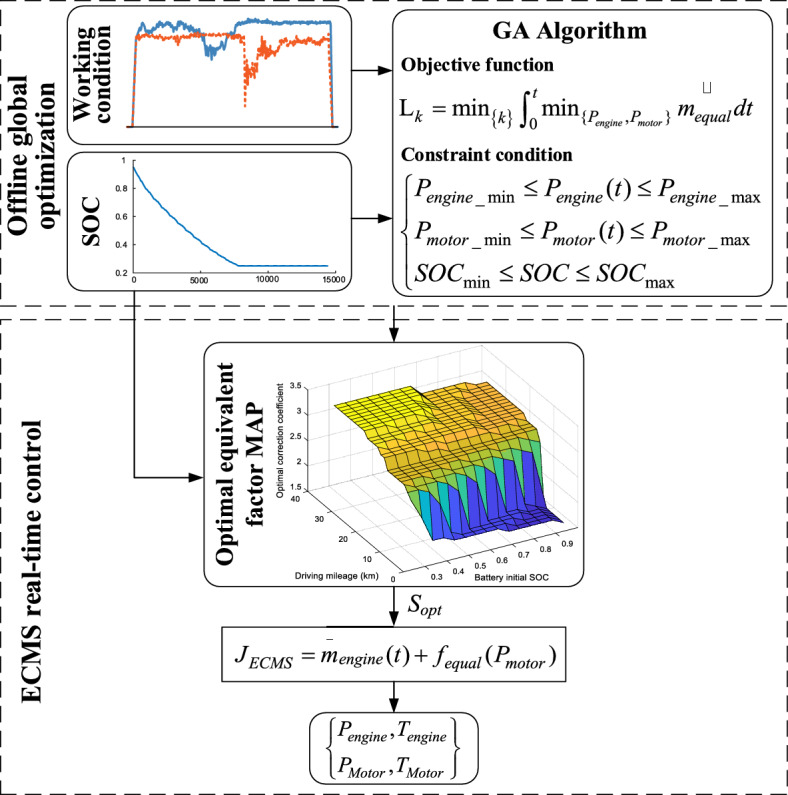
S_opt_-ECMS control policy flow.

To improve the fuel economy of the plug-in hybrid tractor, under the premise of meeting the demand power ${P}_{req}(t)$ at any time in the cycle, using the optimal equivalent factor ${S}_{opt}$ optimization model above, combined with the transmission characteristics of its HMCVT, the S_opt_-ECMS real-time optimization control strategy is established, and the process is shown in Fig. 5. This control strategy can be divided into two parts: global offline optimization correction factor k and ECMS real-time control strategy the modified equivalent factor. In order to prevent the overcharge or overdischarge of the battery caused by the failure of the ECMS strategy, the real-time optimization strategy also includes some rule-based controls, including: when the battery SOC is greater than 0.95, disable the motor charging function to prevent battery overcharge; when the battery SOC is less than 0.15, prohibit the motor power output to prevent battery overdischarge and so on.

The objective function of S_opt_-ECMS real-time optimization control strategy and constraints of the system are changed to the follows.

6 $$\left\{ \begin{gathered} J_{ECMS} = \mathop m\limits_{engine} (t) + f_{equal} (P_{motor} ) \hfill \\ P_{req} (t) = P_{motor} (t) + P_{engine} (t) \hfill \\ P_{engine\_\min } \le P_{engine} (t) \le P_{engine\_\max } \hfill \\ P_{motor\_\min } \le P_{motor} (t) \le P_{motor\_\max } \hfill \\ SOC_{\min } \le SOC \le SOC_{\max } \hfill \\ \end{gathered} \right.$$

Under the premise of satisfying the constraint conditions and power requirements, all power points of the diesel engine and battery are obtained with the required power ${P}_{req}(t)$ at any time, and the fuel consumption rate of the diesel engine and the equivalent fuel consumption rate of the motor are calculated by interpolation from the model of the diesel engine and the motor. Then the minimum equivalent fuel consumption rate is obtained by the objective function in Eq. (5). The working point corresponding to the calculated minimum equivalent fuel consumption of the diesel engine and the motor is taken as the power output at the current moment.

## Verification and analysis

### Comparative analysis before and after optimization

**Figure 6:**
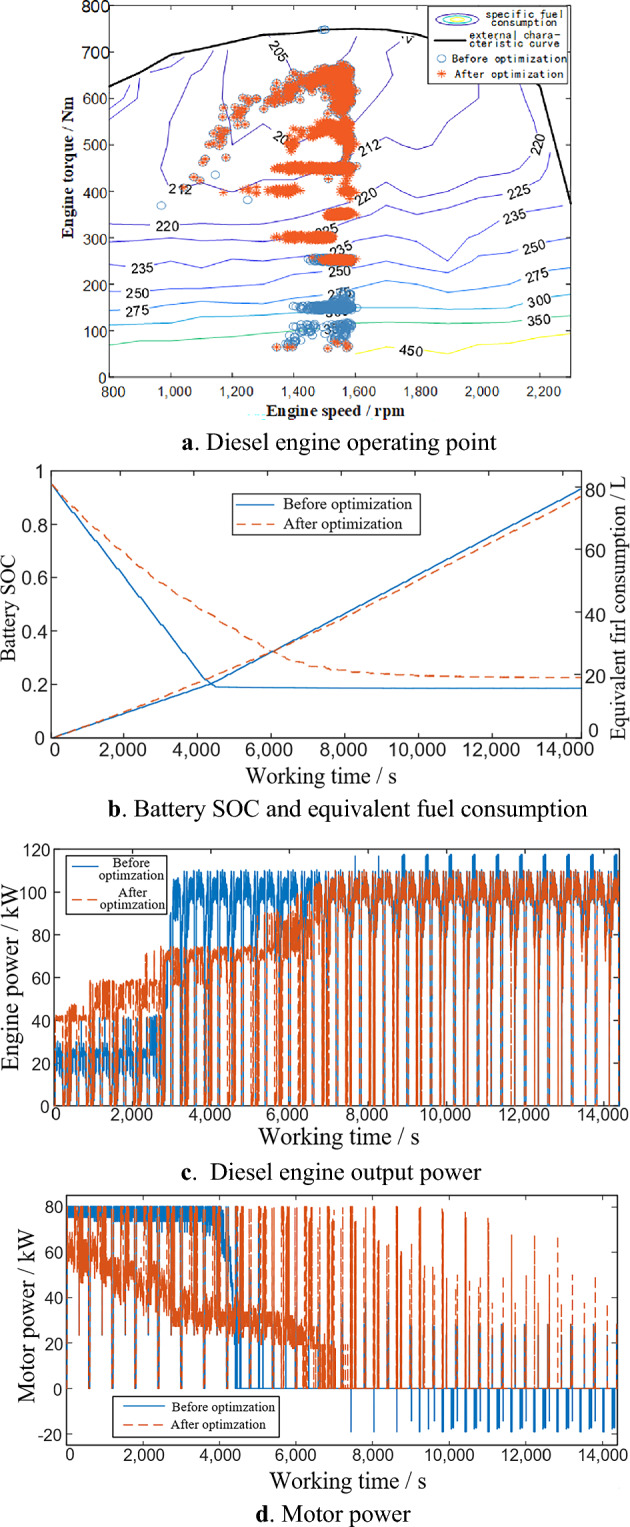
Simulation result.

In order to verify the control effect of the control strategy established above, initial value of the battery SOC is set to 0.95, the simulation working condition is 24 cycles under the working conditions set in the section "Working condition setting" and the simulation time is 14400 s (equivalent to half a working day's working time). Through the simulation test under the above settings, the simulation results shown in the Fig. 6 can be obtained. The diesel engine operating point before and after optimization are shown in the Fig. 6.a, after the optimization of the equivalent factor, the proportion of the working point of the diesel engine in the whole operating cycle appears in the high efficiency area or near the economic optimal curve of the diesel engine is higher. Before and after optimization, the change of battery SOC and equivalent fuel consumption are shown in the Fig. 6.b. After the optimization of the equivalent factor, the battery SOC decreases more slowly, and its final value is closer. and the equivalent fuel consumption is lower at the end of the whole operation cycle. Throughout the operating cycle, the output power of diesel engine and motors at each time is shown in the Figs. 6c, d, and the optimized equivalent factor enables the motor to better assist the diesel engine for efficient output during the whole operation cycle.

Before optimization, due to the small correction coefficient k, in the CD stage, the diesel engine does less work and the motor does more work, which leads to the rapid decline of battery SOC, which leads to the early entry into CS mode. In the CS stage, the motor mainly provides assistance to the engine in the start-up stage and charges the battery when the engine demand torque is obviously lower than the economic optimal curve, resulting in a low proportion of the diesel engine operating point in the high efficiency area. After optimization, the correction coefficient k becomes larger, which makes the diesel engine work more in the CD stage. Although this makes the equivalent fuel consumption at the beginning of operation higher than that before optimization, the decline rate of SOC of the battery slows down obviously, so that the motor can better assist the output of the diesel engine in the whole working cycle, so that the working point of the diesel engine is more concentrated in the high efficiency area, and finally the comprehensive energy consumption is reduced.

From the perspective of the whole operation cycle, the equivalent fuel consumption of hybrid tractors before and after optimization is 79.6901L and 77.3477L respectively, and the final SOC values of batteries before and after optimization are 0.1855 and 0.2261 respectively. Compared with before optimization, the fuel economy after optimization is improved by 2.939%. Therefore, the control strategy proposed in this paper can improve the fuel economy of hybrid tractors and reduce their operating costs.

### Verified by HIL test

**Figure 7:**
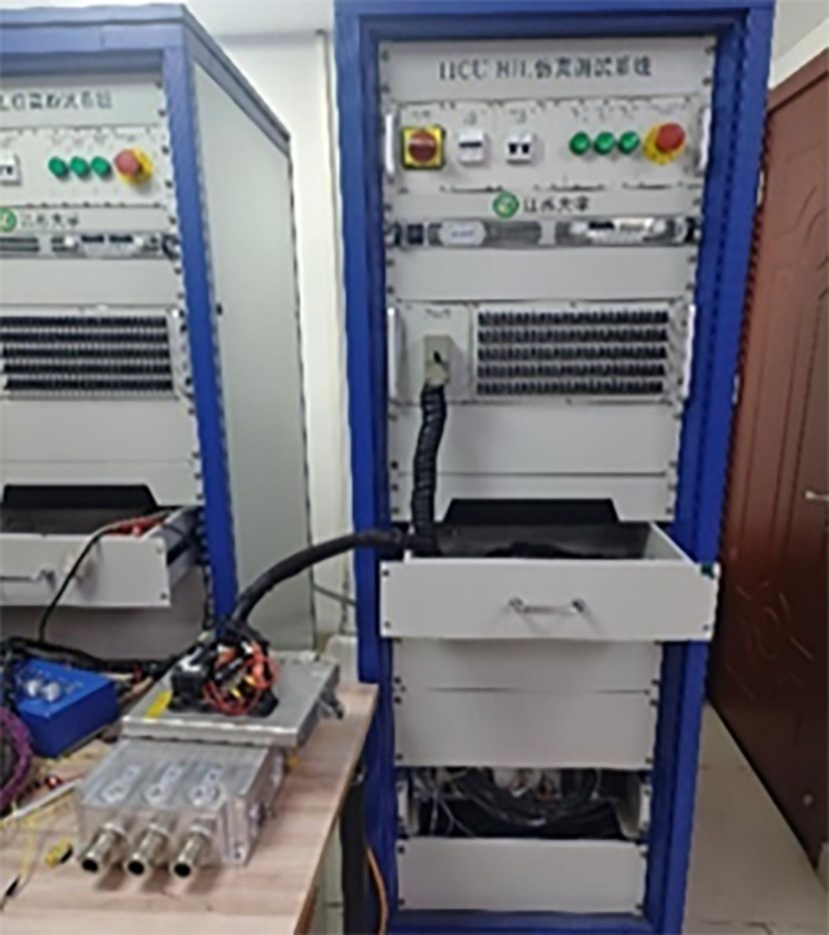
HIL equipment.

In order to verify the effectiveness of the above control strategy, HIL tests are carried out on the equipment shown in Fig. 7. The test cabinet adopts standard shelf design, which mainly includes power management module, programmable power supply, NI real-time simulator, signal conditioning box, load simulation box, distribution box and so on.

The process of HIL testing can be divided into the following three parts:

1. Compilation of controlled object model and construction of test environment

The NIVeriStandSignalProbe module is added to the top layer of the model of the controlled object in Simulink, and the input interface and output interface which need to be mapped in the model are replaced by NIVeriStandIn module and NIVeriStandOut module respectively. Use Matlab RTW to compile the adjusted Simulink model into C code, then use VC to compile the compiled C code into dll file, and then add the dll file to NIVeriStand. The IP address of the host computer is set to be consistent with the RT Target in VeriStand, and devices such as NI DAQ, NI R and reflective memory card are imported into VeriStand.

2. Construction and compilation of controller model

Moto Hark and Simulink model library are used to build the finite time controller model and set up the I/O interface. Then the gcc-powerpc-eabi compiler is used to compile the control algorithm. Finally, the generated algorithm code is added to the vehicle controller using Moto Tune.

3. CAN message communication through I/O port

After importing the interface definition file into the NI XNET device in the HIL test system, add the CAN port to the NI XNET and set the parameters of the CAN port and add specific messages that need to be sent and received through the CAN port. The interfaces are then mapped and matched, and messages are sent periodically from the CAN port.

In the CAN message, the main output signals of the plant model include: engine torque feedback is output at the 0 × 0 address with a signal named "EngTrq" in a step size of 0.01 s, with a value range of [0, 800]; engine speed feedback is output at the 0 × 0 address with a signal named "EngSpd" in a step size of 0.01 s, with a value range of [800, 2300]; motor speed feedback is output at 0 × 0 address with a signal named "MotSpd" with a step size of 0.01 s, with a value range of [− 11000, 11000]; motor torque feedback is output at 0 × 0 address with a signal named "MotTrq" in a step size of 0.01 s, with a value range of [− 30, 80]; the current SOC of the battery is output at the 0 × 1 address with a signal named "SOC" in a step size of 0.01 s, with a value range of [0.1, 1].

The main input signals of the plant model include: engine torque request is output at the 0 × 3 address with a signal named "EngTrq_Req" in a step size of 0.01 s, with a value range of [0,1]; motor torque request is output at the 0 × 3 address with a signal named "MotTrq_Req" in a step size of 0.01 s, with a value range of [0,1]; brake signal is output at the 0 × 3 address with a signal named "BrkSign" in a step size of 0.01 s, with a value range of [0,1]; the initial SOC of the battery is output at the 0 × 3 address with a signal named " SOC_Initial " in a step size of 0.01 s, with a value range of [0.1,1].

**Figure 8:**
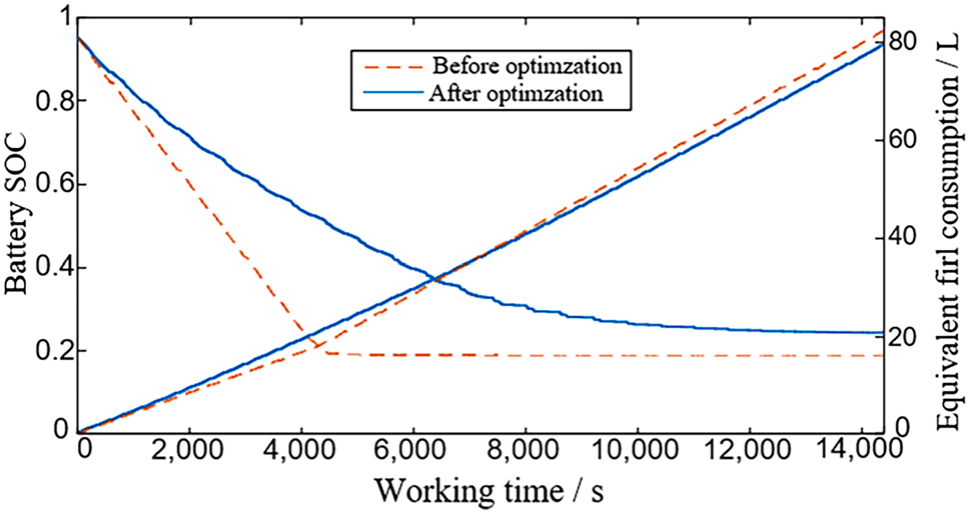
HIL test results.

Calculated based on the HIL test results, the changes in battery power and equivalent fuel consumption of PHET before and after optimization are shown in Fig. 8, the equivalent fuel consumption of the hybrid tractor before and after optimization is 82.413 L and 79.312 L, respectively, and the final SOC values of the battery are 0.188 and 0.242, respectively. In the HIL test, the use of the optimized control strategy leads to a reduction of the equivalent fuel consumption of the hybrid tractor by 3.1004 L and an improvement in fuel economy by 3.909%.

Since the operation of the powertrain components during the HIL cabinet operation will cause the dynamic change of the additional equivalent fuel consumption, it will lead to some deviations between the results of the HIL test and the simulation results in MATLAB/Simulink, but the results are in basic agreement with the simulation results of the model in MATLAB/Simulink. Therefore, through the HIL test, it is further verified that the established energy management control strategy developed in this paper can improve the fuel economy of the plug-in hybrid tractor during longer continuous operation.

## Conclusion

1. A set of hydro-mechanical CVT for plug-in hybrid tractor is designed according to the operational requirements of the tractor, and the efficiency surface of the diesel engine, the efficiency surface of the electric motor, and the optimal economic speed ratio surface of the hydro-mechanical transmission are determined through bench tests. A tillage experiment with a tillage depth of 0.26 m was carried out, and the traction resistance of the tractor during ploughing was measured by tension sensor, and the cycle condition of the tractor was determined by combining the test condition of DLG and the measured traction resistance.
2. According to the working condition of the tractor, the genetic algorithm is used to search the equivalent coefficient of the plug-in hybrid tractor, and the optimal equivalent factor MAP under different the initial value of battery SOC and driving mileage is obtained, and then the ECMS strategy based on equivalent factor optimization is established. The optimized equivalent factor is used to optimize the ECMS in real time, which makes plug-in hybrid tractor in the CD stage as far as possible on the premise of making full use of the energy in the battery and realizes the more effective use of the energy stored in the battery to drive plug-in hybrid tractor in the whole operation cycle.
3. The effectiveness of the control strategy is verified by MATLAB/Simulink simulation experiments and hardware-in-the-loop simulation experiments. The results show that the optimization of equivalent factor optimization can improve the fuel economy of plug-in hybrid tractor by 2.939% in MATLAB/Simulink simulation and 3.909% in hardware-in-the-loop test. The data of the two tests confirm each other, which further verifies that the control strategy proposed in this paper can effectively improve the fuel economy of hybrid tractors.
4. In summary, the equivalent factor global optimization ECMS energy management strategy has certain feasibility and effectiveness in real-time optimization. which can provide a theoretical reference for further solving the adaptive energy management and allocation under different operating conditions. In the future, the neural network and deep learning methods can be used to predict the working time and power of the tractor. According to the predicted working time and the ratio of the predicted power to the reference power after a single replenishment, combined with the optimal equivalent factor MAP, the fuel economy of the plug-in hybrid tractor can be further improved.

## Data Availability

The datasets used and/or analysed during the current study available from the corresponding author on reasonable request.
